# Assessing mammographic density change within individuals across screening rounds using deep learning–based software

**DOI:** 10.1117/1.JMI.12.S2.S22017

**Published:** 2025-08-13

**Authors:** Jakob Olinder, Daniel Förnvik, Victor Dahlblom, Viktor Lu, Anna Åkesson, Kristin Johnson, Sophia Zackrisson

**Affiliations:** a Lund University, Department of Translational Medicine, Radiology Diagnostics, Malmö, Sweden; b Skåne University Hospital, Department of Imaging and Physiology, Malmö, Sweden; c Lund University, Department of Translational Medicine, Medical Radiation Physics, Malmö, Sweden; d Skåne University Hospital, Department of Haematology, Oncology and Radiation Physics, Lund, Sweden; e Skåne University Hospital, Clinical Studies Sweden-Forum South, Lund, Sweden

**Keywords:** breast density, mammography, breast cancer screening, longitudinal trends, breast cancer risk, deep learning

## Abstract

**Purpose:**

The purposes are to evaluate the change in mammographic density within individuals across screening rounds using automatic density software, to evaluate whether a change in breast density is associated with a future breast cancer diagnosis, and to provide insight into breast density evolution.

**Approach:**

Mammographic breast density was analyzed in women screened in Malmö, Sweden, between 2010 and 2015 who had undergone at least two consecutive screening rounds <30 months apart. The volumetric and area-based densities were measured with deep learning–based software and fully automated software, respectively. The change in volumetric breast density percentage (VBD%) between two consecutive screening examinations was determined. Multiple linear regression was used to investigate the association between VBD% change in percentage points and future breast cancer, as well as the initial VBD%, adjusting for age group and the time between examinations. Examinations with potential positioning issues were removed in a sensitivity analysis.

**Results:**

In 26,056 included women, the mean VBD% decreased from 10.7% [95% confidence interval (CI) 10.6 to 10.8] to 10.3% (95% CI: 10.2 to 10.3) (p<0.001) between the two examinations. The decline in VBD% was more pronounced in women with initially denser breasts (adjusted β=−0.10, p<0.001) and less pronounced in women with a future breast cancer diagnosis (adjusted β=0.16, p=0.02).

**Conclusions:**

The demonstrated density changes over time support the potential of using breast density change in risk assessment tools and provide insights for future risk-based screening.

## 1 Introduction

High mammographic breast density increases the risk of breast cancer and also decreases the sensitivity and specificity of mammography in breast cancer screening^1^^–^^3^ with breast cancer arising predominantly from areas of dense tissue.^4^ Previous studies have shown that breast density decreases with increasing age^5^^–^^12^ with a major impact of menopause.^7^^,^^9^^,^^11^^,^^13^^–^^15^ The rate of breast density change is also influenced by other factors, including the initial level of breast density, use of hormonal therapy, ethnicity, and the breast density assessment method.^5^^–^^11^^,^^13^^,^^14^ Studies have shown that a substantial change in breast density, or an increase in the Breast Imaging-Reporting and Data System (BI-RADS) density classification, is associated with future breast cancer,^16^^–^^18^ as demonstrated in a previous meta-analysis in which an increase or decrease in breast density was associated with a higher and a lower risk of future breast cancer, respectively.^19^ However, these studies did not investigate the association between the rate at which breast density changes and the risk of breast cancer. In contrast to several previous studies,^6^^,^^10^^,^^11^ recent evidence suggests an association between the rate of breast density decline and future breast cancer.^20^

Several continuous assessment methods on digital mammography (DM) images have been introduced as alternatives to the BI-RADS density classification,^21^ which is assessed subjectively by radiologists. The proportion of women with dense breasts varies from 6.3% to 84.5% across different radiologists using the fourth edition BI-RADS, with substantial variation in intra-individual agreement (κ=0.21 to 0.79).^22^^,^^23^ In contrast, with the fifth edition BI-RADS, substantial to almost perfect inter-reader agreement has been reported (κ=0.79).^24^ However, other studies show moderately strong agreement, similar to the fourth edition (κ=0.73 versus κ=0.71).^25^ Automatic and continuous methods can overcome the potential risk of inter- and intra-individual disagreement associated with BI-RADS and can also address the nuances created by overlapping tissues with continuous rather than categorical data.^26^ Both area-based and volumetric density assessment tools have been developed, with semiautomatic, automatic, and recently deep learning–based software.^27^^–^^33^

The emerging evidence suggesting breast density change to be a risk factor for subsequent breast cancer raises the possibility of incorporating this variable into risk assessment tools.^20^^,^^34^ Information about the change in breast density over time is thus crucial, and data from population-based screening is needed. Published data on volumetric breast density percentage (VBD%) change from some large screening populations exists,^8^ but it remains limited, especially with regard to changes across different breast density categories. Moreover, with new deep learning–based software to measure breast density, new evaluations are needed. A detailed understanding of breast density evolution is also necessary to enable simulations of realistic changes in digital breast phantoms to be used in virtual clinical trials.^35^ Furthermore, the information about the change in breast density over time is also important when screening strategies based on breast density stratification^36^^,^^37^ or other risk factors are implemented as it holds significance both for the individual woman and for the prediction of future screening workflows.

We aimed to evaluate the change in mammographic density within individuals across screening rounds, using deep learning–based software and fully automated density measurement software. We also aimed to evaluate whether a change in breast density is associated with a future breast cancer diagnosis.

## 2 Method

### 2.1 Participants

**Fig. 1:**
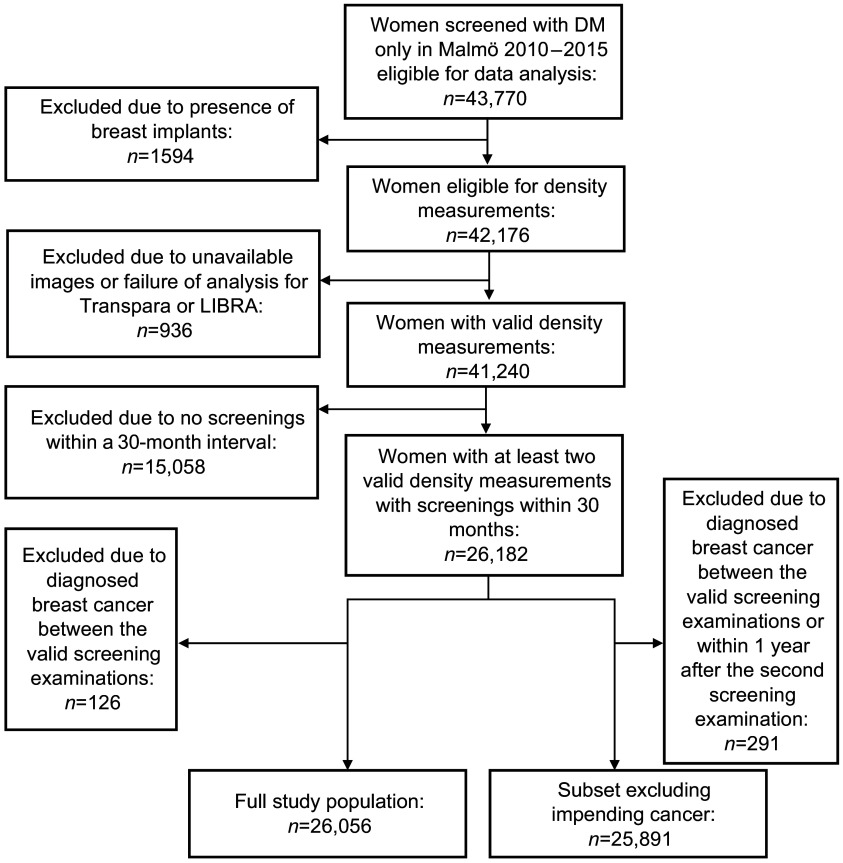
Flowchart of study inclusion. Flowchart of population in the study.

The study population in this retrospective study consisted of all women who attended breast cancer screening in Malmö, Sweden, during the period 2010 to 2015, who were included in the Malmö Breast Imaging (M-BIG) database^38^ and who had not been included in the Malmö Breast Tomosynthesis Screening Trial.^39^ Women who underwent at least two DM screening rounds <30 months apart were included in the analysis. If a woman had several screening rounds <30 months apart, the first two available screening rounds in the study period were analyzed. The first two examinations were used to capture early breast density change information before a potential cancer diagnosis. Women with breast implants and women with a reported breast cancer diagnosis between the two screening rounds were excluded (Fig. 1). Women with previous breast cancer were included if they had re-entered the screening program. The project was conducted in accordance with the Swedish Ethics Review Act (2003:460), and ethical approval was obtained from the ethics review authority (Dnr 2018/322, 2021-00769), and informed consent was waived.

### 2.2 Screening Setting

Over 91% of the DM images in the study population were acquired with Mammomat Inspiration mammographic equipment made by Siemens Healthineers (Erlangen, Germany), whereas a small proportion were obtained with Mammomat Fusion and Mammomat Novation DR equipment from the same manufacturer. All images were for presentation images, as raw images were not routinely saved. Women aged 40 to 74 years were invited every 18 to 24 months, depending on age (18 months for women aged 40 to 54 and 24 months for women aged 55 to 74), in accordance with the Swedish screening recommendations. The DM examinations were double-read with a consensus discussion if required by one or both readers. Breast density is not assessed within the screening program. The maximum 30-month interval between examinations was chosen to ensure that density changes were measured between two consecutive examinations while minimizing the risk of including women who missed a screening round. For women under 54, missing a screening round would result in a gap of at least 36 months (18+18 months).

### 2.3 Breast Density Assessment

VBD% was assessed with the commercial AI software Transpara 2.0.0 (ScreenPoint Medical, Nijmegen, the Netherlands),^33^^,^^40^ from the for-presentation DM images. Using deep learning technology, the AI system calculates the VBD% as a continuous percentage value for each breast and presents the densest recording from the examination as the exam-level VBD% value. The Transpara density software is cleared by the U.S. Food and Drug Administration (FDA) with compatibility shown for mammographic images from Siemens.^41^ The VBD% change of the exam-level breast density between two screening examinations, in percentage points (pp), was computed. The AI system also categorized each breast into a density grade (A to D) in accordance with the American College of Radiology BI-RADS fifth edition.^21^ The highest density grade of the two breasts was considered the exam-level density grade.

The continuous area-based breast percentage density (PD) was assessed with the fully automated software Laboratory for Individualized Breast Radiodensity Assessment (LIBRA) 1.0.5 beta,^28^^,^^29^ from the for-presentation DM images. LIBRA assesses the total breast area and dense area per projection. For presentation, image assessment with LIBRA, as used in this study with the 1.0.5 Beta version, has previously demonstrated a strong association with radiologists’ density assessments (r=0.89) in Siemens Mammomat Inspiration images.^42^ The mean area-based breast percentage density (area-based PD) per examination with LIBRA was calculated as a proportion of the mean dense area of the mean total breast area. The average area-based PD from all available images, using both craniocaudal (CC) and mediolateral oblique (MLO) views of both breasts, was analyzed in accordance with the use in the previous study.^42^ The area-based PD change between two screening examinations, in pp, was computed.

### 2.4 Future Breast Cancer Diagnosis

Any breast cancer diagnosis, screen-detected or non-screen-detected, reported at any time after the second screening round, was collected from the M-BIG database.^43^ The database included breast cancers reported to the register up until May 16, 2022. Cancer cases were ascertained through record matching with the regional cancer registry as well as the National Quality Registry for Breast Cancer, with >98% breast cancer coverage, indicating a low risk of loss to follow-up.^44^ If several cancer cases for the same woman were reported to the registers, we investigated the association with the first cancer diagnosis date reported to any of the two registers at any time after the first included screening examination. All breast cancers reported to the registries were included, both *in situ* and invasive cancer. The median follow-up time was calculated as the median time between the second screening examination up until May 16, 2022. Additional analysis was performed excluding women diagnosed with breast cancer within the first year of the second screening round to exclude capturing of visible breast tumor tissue of screen-detected cancers as a reason for the density change.

### 2.5 Age Groups

Menopausal status was not recorded in the medical records. Women were divided into non-uniform age groups to account for the known effect of menopausal status on breast density changes.^7^^,^^9^^,^^11^^,^^13^^–^^15^ The age groups were 40 to 45 years to include most women before the onset of perimenopause, 45 to 54 years to include most women in the menopausal transition, and 55 years and older to include most postmenopausal women.^45^^,^^46^

### 2.6 Management of Different Positioning

To adjust for the possibility of different breast positioning and compression, resulting in non-biological variability between screening examinations,^47^ a separate sensitivity analysis was performed. A large change in breast area was used as a proxy for differences in breast positioning. Using the results from LIBRA, we excluded women with more than a 25% change in breast area between the two screening rounds in the sensitivity analysis (Figs. S1 and S2 in the Supplementary Material). To the best of our knowledge, no previous studies have investigated the normal variation of area change across screening examinations with LIBRA. The sensitivity analysis, which excluded women with more than a 25% change in breast area, was a *post hoc* analysis selected arbitrarily for practical reasons. Most women had an area change of <25% which was considered to be a normal variation in the material after analysis of the area change distribution (Fig. S3 in the Supplementary Material). The results prior to the sensitivity analysis reflect real-world screening data and were therefore presented as the main findings.

### 2.7 Language Editing

ChatGPT (versions 3.5 and 4.0) was used as a language editing tool for text and script for the R statistical software. The authors are creators of all the intellectual content; none of the intellectual content or information was originally created by the large language model.

### 2.8 Statistical Analysis

Breast density changes, in pp, within individuals between the second and the first screening rounds were calculated for the two breast density assessment methods (exam-level VBD% with Transpara and average area-based PD with LIBRA) with 95% confidence intervals (CIs), calculated using Student’s t-distribution. Breast density was compared with a paired t-test between the second and first included screening rounds. The participants were stratified into groups according to categorical density from Transpara (A to D) at the first screening round, whether the woman had a future breast cancer diagnosis after the second screening round (with additional analysis performed to exclude women diagnosed with breast cancer within the first year after the second screening round), and non-uniform age groups. Multiple linear regression was performed, using the exam-level VBD% change in pp between two screening examinations as the dependent variable to investigate its association with future breast cancer diagnosis, as well as the initial VBD% with adjustments for non-uniform age groups and time between examinations (in months). Additional multiple linear regression of factors associated with VBD% change in pp was performed after the exclusion of women diagnosed with breast cancer within the first year after the second screening round. For completeness, multiple linear regression was also performed to investigate the association between the area-based PD change in pp between two screening examinations with the same factors. All the unstratified analyses and multiple linear regression were also performed after the exclusion of cases with potential positioning issues in the sensitivity analysis. Statistical analyses were performed using the R software (version 4.3.2, R Foundation for Statistical Computing, Vienna, Austria), with an alpha level of 0.05 applied for significance testing. A Bonferroni correction for multiple hypothesis testing was applied to the paired t-tests, adjusting for 40 comparisons. These included comparisons across two methods (Transpara and LIBRA), with nine stratifications and one total and with tests both for all cancers as well as for those diagnosed at least 1 year after the second screening round. The significance threshold was, after correction, set to α=0.00125.

## 3 Results

### 3.1 Participant Characteristics

The study included 26,056 women out of 43,770 women in the database. The exclusions were due to breast implants (n=1594), failure of software density analysis or unavailability of images (n=936), women without two screenings within 30 months apart (n=15,098), and women with breast cancer diagnosed between the two screening rounds (n=126). A breast cancer diagnosis at any time after the second screening round was reported for 891 women. Data for age, days between examinations, menopausal status, future breast cancer diagnosis, median follow-up time in months, BI-RADS density categories, area-based PD, and VBD% from the first included screening examination are presented in Table 1, along with additional analysis after exclusion of women diagnosed with breast cancer within 1 year after the second screening examination (n=165).

**Table 1:** Descriptive data of the study population.

	Full study population	Subset excluding impending cancer
	Mean (SD) or number (%)	Mean (SD) or number (%)
Total number of participants	26,056 (100%)	25,891 (100%)
Age at first screening	52.7 years (9.5)	52.7 years (9.5)
Days between examinations	659 days (109)	659 days (109)
Age groups		
Age 40 to 44	7097 (27.2%)	7072 (27.3%)
Age 45 to 54	8887 (34.1%)	8843 (34.2%)
Age 55 to 74	10,072 (38.7%)	9976 (38.5%)
Future cancer		
Yes	891 (3.4%)	726 (2.8%)
No	25,165 (96.6%)	25,165 (97.2%)
Median follow-up time	111 months (20)	111 months (20)^a^
BI-RADS density category at first screening		
A	2506 (9.6%)	2492 (9.6%)
B	10,740 (41.2%)	10,674 (41.2%)
C	9012 (34.6%)	8954 (34.6%)
D	3798 (14.6%)	3771 (14.6%)
Area-based PD at first screening	27.7% (16.5)	27.7% (16.5)
VBD% at first screening	10.7% (5.4)	10.7% (5.4)

Descriptive data based on the first included screening examination are presented for the full study population and for the subset excluding women with impending cancer diagnosed up to 1 year after the second screening examination (additional analysis).

a Follow-up time (months) presented as median with interquartile range.

### 3.2 Breast Density Change

The mean values of VBD% and area-based PD decreased from 10.7% (95% CI: 10.6 to 10.8) to 10.3% (95% CI: 10.2 to 10.3) (p<0.001) and from 27.7% (95% CI: 27.5 to 27.9) to 26.3% (95% CI: 26.1 to 26.5) (p<0.001), respectively (Table 2). The corresponding values from the analysis when women diagnosed with breast cancer up to 1year after the second screening examination were excluded are presented in Table 3. The mean time between screening rounds was ∼22 months in both analyses.

**Table 2:** Breast density change over one screening interval.

	Days between examinations (95% CI)	Density on first examination (95% CI)	Density on second examination (95% CI)	Density change in pp (95% CI)	p-value using paired t-test
Area-based PD	659 (658, 661)	27.7 (27.5, 27.9)	26.3 (26.1, 26.5)	−1.4(−1.4, −1.3)	<0.001
VBD%	659 (658, 661)	10.7 (10.6, 10.8)	10.3 (10.2, 10.3)	−0.4 (−0.5, −0.4)	<0.001
Density					
Density A					
Area-based PD	697 (693, 701)	10.3 (10.2, 10.4)	10.1 (10.0, 10.2)	−0.1 (−0.2, −0.0)	0.014
VBD%	697 (693, 701)	4.9 (4.8, 4.9)	5.1 (5.1, 5.1)	0.2 (0.2, 0.3)	<0.001
Density B					
Area-based PD	679 (677, 682)	16.6 (16.5, 16.6)	15.9 (15.8, 16.0)	−0.7 (−0.7, −0.6)	<0.001
VBD%	679 (677, 682)	7.2 (7.2, 7.2)	7.0 (7.0, 7.1)	−0.2 (−0.2, −0.2)	<0.001
Density C					
Area-based PD	641 (639, 643)	34.0 (33.8, 34.3)	32.0 (31.8, 32.2)	−2.0 (−2.2, −1.9)	<0.001
VBD%	641 (639, 643)	12.1 (12.0, 12.1)	11.5 (11.4, 11.6)	−0.6 (−0.6, −0.5)	<0.001
Density D					
Area-based PD	622 (619, 625)	55.4 (55.1, 55.8)	52.8 (52.5, 53.2)	−2.6 (−2.9, −2.3)	<0.001
VBD%	622 (619, 625)	21.1 (21.0, 21.2)	19.8 (19.7, 20.0)	−1.3 (−1.3, −1.2)	<0.001
Future cancer					
Yes					
Area-based PD	672 (664, 680)	29.2 (28.1, 30.3)	28.1 (27.0, 29.1)	−1.1 (−1.5, −0.8)	<0.001
VBD%	672 (664, 680)	11.4 (11.0, 11.8)	11.0 (10.7, 11.4)	−0.4 (−0.5, −0.2)	<0.001
No					
Area-based PD	659 (658, 660)	27.6 (27.4, 27.8)	26.2 (26.0, 26.4)	−1.4 (−1.4, −1.3)	<0.001
VBD%	659 (658, 660)	10.7 (10.6, 10.7)	10.2 (10.2, 10.3)	−0.4 (−0.5, −0.4)	<0.001
Age groups					
Age 40 to 44					
Area-based PD	609 (608, 610)	36.9 (36.5, 37.3)	35.6 (35.2, 36.0)	−1.3 (−1.5, −1.1)	<0.001
VBD%	609 (608, 610)	13.4 (13.3, 13.6)	13.0 (12.8, 13.1)	−0.4 (−0.5, −0.4)	<0.001
Age 45 to 54					
Area-based PD	595 (594, 597)	29.1 (28.8, 29.5)	27.1 (26.8, 27.5)	−2.0 (−2.1, −1.9)	<0.001
VBD%	595 (594, 597)	11.0 (10.9, 11.1)	10.4 (10.3, 10.5)	−0.7 (−0.7, −0.6)	<0.001
Age 55 to 74					
Area-based PD	751 (749, 753)	19.9 (19.6, 20.1)	19.0 (18.8, 19.2)	−0.9 (−0.9, −0.8)	<0.001
VBD%	751 (749, 753)	8.5 (8.4, 8.5)	8.2 (8.1, 8.3)	−0.2 (−0.3, −0.2)	<0.001

Mean area-based breast PD and VBD% from the first and second included screening examinations. The table includes the number of days between examinations and the difference in density, in pp, between them. Results are stratified by BI-RADS fifth edition, obtained from Transpara; by future breast cancer diagnosis after the second screening examination; and by age groups. CI using Student’s t-distribution.

**Table 3:** Breast density change over one screening interval for the subset excluding women with impending cancer diagnosed up to 1 year after the second screening examination.

	Days between examinations (95% CI)	Density on first examination (95% CI)	Density on second examination (95% CI)	Density change in pp (95% CI)	p-value using paired t-test
Area-based PD	659 (658, 660)	27.7 (27.5, 27.9)	26.3 (26.1, 26.5)	−1.4(−1.4, −1.3)	<0.001
VBD%	659 (658, 660)	10.7 (10.6, 10.8)	10.3 (10.2, 10.3)	−0.4 (−0.5, −0.4)	<0.001
Density					
Density A					
Area-based PD	697 (692, 701)	10.3 (10.2, 10.4)	10.1 (10.0, 10.2)	−0.1 (−0.2, −0.0)	0.017
VBD%	697 (692, 701)	4.9 (4.8, 4.9)	5.1 (5.1, 5.1)	0.2 (0.2, 0.3)	<0.001
Density B					
Area-based PD	679 (677, 681)	16.6 (16.5, 16.6)	15.9 (15.8, 16.0)	−0.7 (−0.7, −0.6)	<0.001
VBD%	679 (677, 681)	7.2 (7.2, 7.2)	7.0 (7.0, 7.1)	−0.2 (−0.2, −0.2)	<0.001
Density C					
Area-based PD	640 (638, 643)	34.1 (33.8, 34.3)	32.0 (31.8, 32.3)	−2.0 (−2.2, −1.9)	<0.001
VBD%	640 (638, 643)	12.1 (12.0, 12.1)	11.5 (11.4, 11.6)	−0.6 (−0.6, −0.5)	<0.001
Density D					
Area-based PD	622 (619, 625)	55.4 (55.1, 55.8)	52.8 (52.5, 53.2)	−2.6 (−2.9, −2.3)	<0.001
VBD%	622 (619, 625)	21.1 (21.0, 21.2)	19.8 (19.7, 20.0)	−1.3 (−1.4, −1.2)	<0.001
Future cancer^a^					
Yes					
Area-based PD	667 (658, 676)	29.6 (28.4, 30.8)	28.3 (27.2, 29.5)	−1.3 (−1.7, −0.9)	<0.001
VBD%	667 (658, 676)	11.6 (11.1, 12.0)	11.2 (10.8, 11.6)	−0.4 (−0.5, −0.2)	<0.001
No					
Area-based PD	659 (658, 660)	27.6 (27.4, 27.8)	26.2 (26.0, 26.4)	−1.4 (−1.4, −1.3)	<0.001
VBD%	659 (658, 660)	10.7 (10.6, 10.7)	10.2 (10.2, 10.3)	−0.4 (−0.5, −0.4)	<0.001
Age groups					
Age 40 to 44					
Area-based PD	609 (607, 610)	36.9 (36.5, 37.3)	35.6 (35.2, 36.0)	−1.3 (−1.5, −1.1)	<0.001
VBD%	609 (607, 610)	13.4 (13.3, 13.6)	13.0 (12.8, 13.1)	−0.4 (−0.5, −0.4)	<0.001
Age 45 to 54					
Area-based PD	595 (594, 597)	29.1 (28.8, 29.4)	27.1 (26.8, 27.4)	−2.0 (−2.1, −1.9)	<0.001
VBD%	595 (594, 597)	11.0 (10.9, 11.1)	10.4 (10.3, 10.5)	−0.6 (−0.7, −0.6)	<0.001
Age 55 to 74					
Area-based PD	751 (749, 753)	19.8 (19.6, 20.1)	19.0 (18.8, 19.2)	−0.9 (−0.9, −0.8)	<0.001
VBD%	751 (749, 753)	8.5 (8.4, 8.5)	8.2 (8.1, 8.3)	−0.2 (−0.3, −0.2)	<0.001

Mean area-based breast PD and VBD% from the first and second included screening examinations, for the subset excluding women with impending cancer diagnosed up to 1 year after the second screening examination. The table includes the number of days between examinations and the difference in density, in pp, between them. Results are stratified by BI-RADS fifth edition, obtained from Transpara.

a Future breast cancer diagnosed at least 1 year after the second screening examination; and by age groups. CI using Student’s t-distribution.

### 3.3 Breast Density Change over Density Categories

A decline in the VBD% was seen for all BI-RADS categories except BI-RADS A, which increased by 0.2 pp (95% CI: 0.2 to 0.3, p<0.001). BI-RADS B, C, and D decreased by 0.2 pp (95% CI: 0.2 to 0.2, p<0.001), 0.6 pp (95% CI: 0.5 to 0.6, p<0.001), and 1.3 pp (95% CI: 1.2 to 1.3, p<0.001), respectively. A decline in the area-based PD was noted for all BI-RADS categories except BI-RADS A 0.1 pp (95% CI: 0.0 to 0.2, p=0.014), where the change was non-significant. BI-RADS B, C, and D decreased by 0.7 pp (95% CI: 0.6 to 0.7, p<0.001), 2.0 pp (95% CI: 1.9 to 2.2, p<0.001), and 2.6 pp (95% CI: 2.3 to 2.9, p<0.001), respectively. The analysis when women diagnosed with breast cancer up to 1 year after the second screening examination were excluded resulted in very similar values.

### 3.4 Initial Breast Density and Breast Density Change

Unadjusted analysis showed that the VBD% change in pp between two screening examinations was significantly associated with the VBD% at the first included screening round. Women with higher initial VBD% showed a larger decline in breast density [β=−0.09 (95% CI: −0.10 to −0.09), p<0.001], replicated in the adjusted models of factors associated with breast density change over two screening examinations [adjusted β=−0.10 (95% CI: −0.10 to −0.10), p<0.001] (Table 4). The same results were achieved in the analysis when women diagnosed with breast cancer up to 1year after the second screening examination were excluded (Table 5).

**Table 4:** Multiple linear regression of volumetric breast density percentage change.

Variable	Unadjusted		Adjusted	
	Estimate (95% CI)	p-value	Estimate (95% CI)	p-value
Future cancer				
Yes	0.07 (−0.07, 0.20)	0.33	0.16 (0.03, 0.29)	0.02
Initial VBD%				
Percentage	−0.09 (−0.10, −0.09)	<0.001	−0.10 (−0.10, −0.10)	<0.001
Age groups				
Age 40 to 44	Reference	—	Reference	—
Age 45 to 54	−0.21 (−0.27, −0.15)	<0.001	−0.46 (−0.52, −0.40)	<0.001
Age 55 to 74	0.20 (0.14, 0.26)	<0.001	−0.18 (−0.25, −0.10)	<0.001
Time between examinations				
Months	0.02 (0.01, 0.02)	<0.001	−0.03 (−0.04, −0.02)	<0.001

Results of a multiple linear regression examining the change in VBD% between two screening examinations used as dependent variable. The model includes future cancer diagnosis, initial VBD%, age groups, and the time between examinations as predictors. Unadjusted data for each independent variable are also presented for comparison.

**Table 5:** Multiple linear regression of volumetric breast density percentage change for the subset excluding women with impending cancer diagnosed up to 1 year after the second screening examination.

Variable	Unadjusted		Adjusted	
	Estimate (95% CI)	p-value	Estimate (95% CI)	p-value
Future cancer^a^				
Yes	0.07 (−0.08, 0.22)	0.35	0.17 (0.03, 0.31)	0.02
Initial VBD%				
Percentage	−0.09 (−0.10, −0.09)	<0.001	−0.10 (−0.10, −0.10)	<0.001
Age groups				
Age 40 to 44	Reference	—	Reference	—
Age 45 to 54	−0.21 (−0.27, −0.15)	<0.001	−0.46 (−0.52, −0.40)	<0.001
Age 55 to 74	0.20 (0.14, 0.26)	<0.001	−0.18 (−0.25, −0.10)	<0.001
Time between examinations				
Months	0.02 (0.01, 0.02)	<0.001	−0.03 (−0.03, −0.02)	<0.001

Results of a multiple linear regression examining the change in VBD% between two screening examinations used as dependent variable, for the subset excluding women with impending cancer diagnosed up to 1 year after the second screening examination. The model includes future cancer diagnosis, initial VBD%, age groups, and the time between examinations as predictors. Unadjusted data for each independent variable are also presented for comparison.

a Future cancer is defined as a cancer diagnosed at least 1 year after the second screening round up until the end of follow-up.

### 3.5 Association Between Breast Density Change and Future Breast Cancer Diagnosis

A decline in the VBD% was seen for women both with and without a future diagnosis of breast cancer, 0.4 pp (95% CI: 0.2 to 0.5, p<0.001) and 0.4 pp (95% CI: 0.4 to 0.5, p<0.001), respectively. Similarly, a decline in the area-based PD was seen for women with and without a future diagnosis of breast cancer, 1.1 pp (95% CI: 0.8 to 1.5, p<0.001) and 1.4 pp (95% CI: 1.3 to 1.4, p<0.001), respectively. In the unadjusted analysis, the VBD% change in pp between two screening examinations was not associated with future breast cancer diagnosis [β=0.07 (95% CI: −0.07 to 0.20), p=0.33]. However, after adjustment of factors associated with breast density change between two screening examinations, a slower decline in density was seen for women who had a future breast cancer diagnosis [adjusted β=0.16 (95% CI: 0.03 to 0.29), p=0.02]. The analysis when women diagnosed with breast cancer up to 1 year after the second screening examination were excluded resulted in very similar values.

### 3.6 Impact of Age on Breast Density Change

A decline in the VBD% was seen for all age groups. Women aged 40 to 44, 45 to 54, and 55 to 74 showed a decrease of 0.4 pp (95% CI: 0.4 to 0.5, p<0.001), 0.7 pp (95% CI: 0.6 to 0.7, p<0.001), and 0.2 pp (95% CI: 0.2 to 0.3, p<0.001), respectively. A decline in the area-based PD was similarly seen for all age groups: women 40 to 44 years, 1.3 pp (95% CI: 1.1 to 1.5, p<0.001); women 45 to 54 years, 2.0 pp (95% CI: 1.9 to 2.1, p<0.001); and women 55 to 74 years, 0.9 pp (95% CI: 0.8 to 0.9, p<0.001). Women aged 45 to 54 had a faster decline in VBD% compared with younger women aged 40 to 44, both in the unadjusted [β=−0.21 (95% CI: −0.27 to −0.15), p<0.001] and the adjusted analysis [adjusted β=−0.46 (95% CI: −0.52 to −0.40), p<0.001]. Women aged 55 to 74 showed a slower absolute decline in the VBD% compared with women aged 40 to 44 in the unadjusted analysis [β=0.20 (95% CI: 0.14 to 0.26), p<0.001]. However, adjusted analysis showed a faster decline in density for women aged 55 to 74 compared with younger women aged 40 to 44 [adjusted β=−0.18 (95% CI: −0.25 to −0.10), p<0.001]. The analysis when women diagnosed with breast cancer up to 1 year after the second screening examination were excluded resulted in very similar values.

### 3.7 Results Concerning Area-Based Breast Percentage Density Change

The analysis of multiple linear regression of change in the area-based PD showed estimated coefficients (β) consistently oriented in the same direction as with the VBD% change. An association was seen between the breast density change and future breast cancer for the full study population [adjusted β=0.50 (95% CI: 0.11 to 0.88), p=0.01], but not in the additional analysis, when women diagnosed with cancer up to 1 year after the second screening round were excluded [adjusted β=0.36 (95% CI: −0.07 to 0.78), p=0.10] (Tables S1 and S2 in the Supplementary Material).

### 3.8 Results After Exclusions Due to Possible Positioning Issues

Six hundred sixty-two women (2.5% of the study population) (660 women in the additional analysis) had a change in breast area of more than 25% between screening rounds and were excluded from the sensitivity analysis. The results of the sensitivity analysis are presented in Figs. S1 and S2 and Tables S3–S9 in the Supplementary Material. After adjustment of factors associated with VBD% change in pp over two screening rounds, the association with future breast cancer diagnosis after the second screening examination and after excluding women diagnosed with breast cancer up to 1 year after the second screening round was similar [adjusted β=0.13 (95% CI: 0.00 to 0.25), p=0.04, and adjusted β=0.14 (95% CI: 0.00 to 0.27), p=0.048, respectively]. Similar results were also seen with the association between area-based PD change in pp and future breast cancer diagnosis after the second screening examination in the sensitivity analysis [adjusted β=0.42 (95% CI: 0.04 to 0.79), p=0.03]. Similar to the full analysis, no association was seen between the area-based PD change in pp and future breast cancer when women diagnosed with cancer up to 1 year after the second screening round were excluded [adjusted β=0.24 (95% CI: −0.17 to 0.66), p=0.25].

## 4 Discussion

Breast density decreased between two consecutive breast cancer screening examinations for women aged 40 to 74, both when measured volumetrically using deep learning–based software and when measured in an area-based manner using fully automated software (0.4 pp, 95% CI: 0.4 to 0.5, p<0.001 and 1.4 pp, 95% CI: 1.3 to 1.4, p<0.001, respectively). The decline was more pronounced in women with dense breasts [adjusted β=−0.10 (95% CI: −0.10 to −0.10), p<0.001]. This study adds evidence regarding the magnitude of change in breast density over one screening round. The study also shows that women who later were diagnosed with breast cancer had a lower rate of decline in the VBD% [adjusted β=0.16 (95% CI: 0.03 to 0.29), p=0.02] with a sustained association when restricting the analysis to breast cancers diagnosed more than 1 year after the last included screening examination [adjusted β=0.17 (95% CI: 0.03 to 0.31), p=0.02].

In this study, examining a large, unselected screening population in a high-participation rate program, and using two independent software tools to measure breast density, we found a similar rate of breast density change as previously reported from a non-cancer-enriched screening population.^8^ The population in that previous study consisted of women aged 50 to 69, and a 0.4 to 1.3 pp reduction in the VBD% over 2 years was reported (depending on body mass index and use of hormonal therapy).^8^ The similar rate of breast density change as in our study indicates the robustness of automatic density assessment tools.

The risk of breast cancer increases with breast density.^1^ A correlation between increasing breast density and breast cancer has also been shown. Among women with a family history of breast cancer, stable breast density over time, compared with more than a 10% annual decline, was associated with future cancer.^16^ Studies have also shown an association between an increase in BI-RADS density category over time and breast cancer.^17^^,^^18^ These associations have been further confirmed in a meta-analysis.^19^ However, the change in breast density required in studies to correspond to a shift in BI-RADS category or to be classified as an increase or decrease may be substantial. Results in the literature have been inconsistent on whether an association exists between the rate of breast density change and breast cancer. A smaller case-control study found an increase in breast density for women with breast cancer.^42^ Jiang et al.^20^ found in 2023 a significantly slower decline in breast density in breasts with future cancer than for controls, as indicated in a previous study.^48^ However, other studies have not found evidence that the breast density change differs substantially between women with or without future breast cancer.^6^^,^^10^^,^^11^^,^^49^ Some of the studies have been case-control studies, measuring area-based breast density using semiautomatic software in only one projection (either CC or MLO).^6^^,^^10^^,^^11^ One of the studies, which used a fully automated density assessment tool with machine learning on a screening cohort, focused on investigating changes in breast density in the contralateral breast (the breast that did not develop cancer).^49^ Also, Jiang et al.^20^ found an association between breast density change and breast cancer in the breast that later developed cancer but no significant association when both breasts were analyzed together. These differences in assessment methods and the choice of only investigating the contralateral breast may account for some of the differences in results compared with our study. Our data support the results found by Jiang et al.,^20^ highlighting the potential of breast density change over time as a factor to incorporate into breast cancer risk assessment tools.

The findings focus on volumetric rather than area-based breast percentage density change. Given the promising results for AI in screening,^50^^,^^51^ clinical implementation would be facilitated by a single software platform capable of performing both breast density assessment and cancer likelihood estimation. Using only one software platform would support streamlined integration into clinical practice, as enabled by the deep learning–based tools. However, the lack of a significant association between change in area-based PD and future cancer beyond 1 year after the second screening round underscores the uncertainty of these results. The metrics used to assess density change (densest recording versus average density) between the volumetric and area-based software may contribute to the discrepancy. Differences may also stem from the software’s ability to manage real-world data, including variations in positioning.

Several studies have investigated the effect of menopause on breast density change over time.^7^^,^^9^^,^^11^^,^^13^^–^^15^ The dataset in this study did not include menopausal status. Women were divided into non-uniform age groups to account for the effects of both age and menopausal status on breast density change. The results should thus be interpreted with caution. Women between 45 and 54 years old, an age group used as a proxy to capture most women in menopausal transition, showed a steeper decline in breast density. Previous studies have shown this steeper decline for women during the menopausal transition. ^7^^,^^9^^,^^11^^,^^13^^–^^15^ A faster decline in breast density was also seen for women aged 55 to 74 even though the absolute decline was slower. This faster breast density reduction has in previous studies been shown to occur post-menopausal rather than pre-menopausal.^13^^,^^15^ Unlike several other studies, our population included women aged 40 to 49, which may result in differences in breast density change compared with other studies. This could be important, as the current recommendation in the United States is to include these women in breast cancer screening.^52^

The more pronounced decline in breast density among women with higher initial breast density, as observed in our study, has also been shown in previous research.^9^^,^^11^^,^^14^ Previous studies also showed that the breast density category changes over time, especially in women with dense breasts.^53^ Screening guidelines recommend different screening methods, such as MRI or digital breast tomosynthesis (DBT) for women with dense breasts,^36^^,^^37^ with varying benefits depending on density level.^54^ Even if the change of breast density between two screening examinations is small, the cumulative effect across multiple rounds may still influence the recommended screening method. Therefore, understanding the average rate of VBD% change in pp in a screening population is important for predicting future screening workflows and informing participants about possible future screening methods. The measurements of breast density change can also be used when developing digital breast phantoms for virtual clinical trials.^35^

### 4.1 Limitations

Our study had limitations. First, it was a retrospective study from a Swedish population-based screening program with Swedish screening intervals, not including data on factors that may affect breast density change, such as ethnicity, BMI, physical activity, or a previous cancer diagnosis. The database did not include hormonal therapy use, although the defined daily dose in Sweden is low, especially during the study period of 2010 to 2015, with ∼50 defined daily doses per 1000 women aged 50 to 54.^55^ In addition, this study only included one screening round, and larger effects might have been seen across multiple rounds. Menopausal status was not recorded in the screening database, and the age of menopausal transition is individual;^45^^,^^46^ thus, the use of non-uniform age grouping is a limitation. Another limitation is the use of a volumetric density measurement from DM images, thus estimating a 3D volume from 2D images. However, with the FDA-cleared Transpara software, density showed a strong correlation of 0.908 (95% CI: 0.878 to 0.931) between volumetric breast density estimates and estimates from breast MRI.^41^ Our study focused solely on DM, used as the primary screening tool in the Swedish screening program, thus limiting the results for DBT used in several countries. Studies have shown a tendency for a lower breast density classification with DBT than DM,^56^ whereas others report substantial agreement in density measurements^57^ and similar κ statistics and downgrading rates over time for both modalities.^58^ Thus, our result may not be applicable to density assessments with DBT. The use of different methods to assess VBD% (the densest recording of the two breasts) and area-based PD (the average density of both breasts) may limit the comparability of density change between the two approaches. However, their alignment with clinically reported values and consistency with previous studies support the validity of each method’s individual results.

## 5 Conclusion

Mammographic breast density, measured continuously using both deep learning–based software and fully automated software, shows a small but statistically significant decrease between two consecutive screening rounds with different decreases depending on the initial breast density category. The decline was more pronounced in women with dense breasts (adjusted β=−0.10, p<0.001). The rate of change is in line with previous studies, indicating the robustness of automatic density assessment tools. Furthermore, a future breast cancer diagnosis is associated with a slower decline in the volumetric breast density percentage (adjusted β=0.16, p=0.02) compared with women without a breast cancer diagnosis. Our data indicate the possibility of incorporating breast density change in risk assessment tools and provide insights into breast density evolution, which are important for both virtual clinical trials and future risk-based screening.

## 6 Supplementary Material

10.1117/1.JMI.12.S2. S22017.s01

## Data Availability

The datasets used in this study are not publicly available because of privacy and ethical restrictions.
