# Supplementary material for: Assessing mammographic density change within individuals across screening rounds using deep learning–based software
Source: J Med Imaging (Bellingham). 2025 Aug 13;12(Suppl 2):S22017. doi: 10.1117/1.JMI.12.S2.S22017 (PMC12350635; doi:10.1117/1.JMI.12.S2.S22017)
Supplement: Supplementary file 1 [file JMI_012_S22017_SD001.pdf]

**Supplemental figures and tables:**

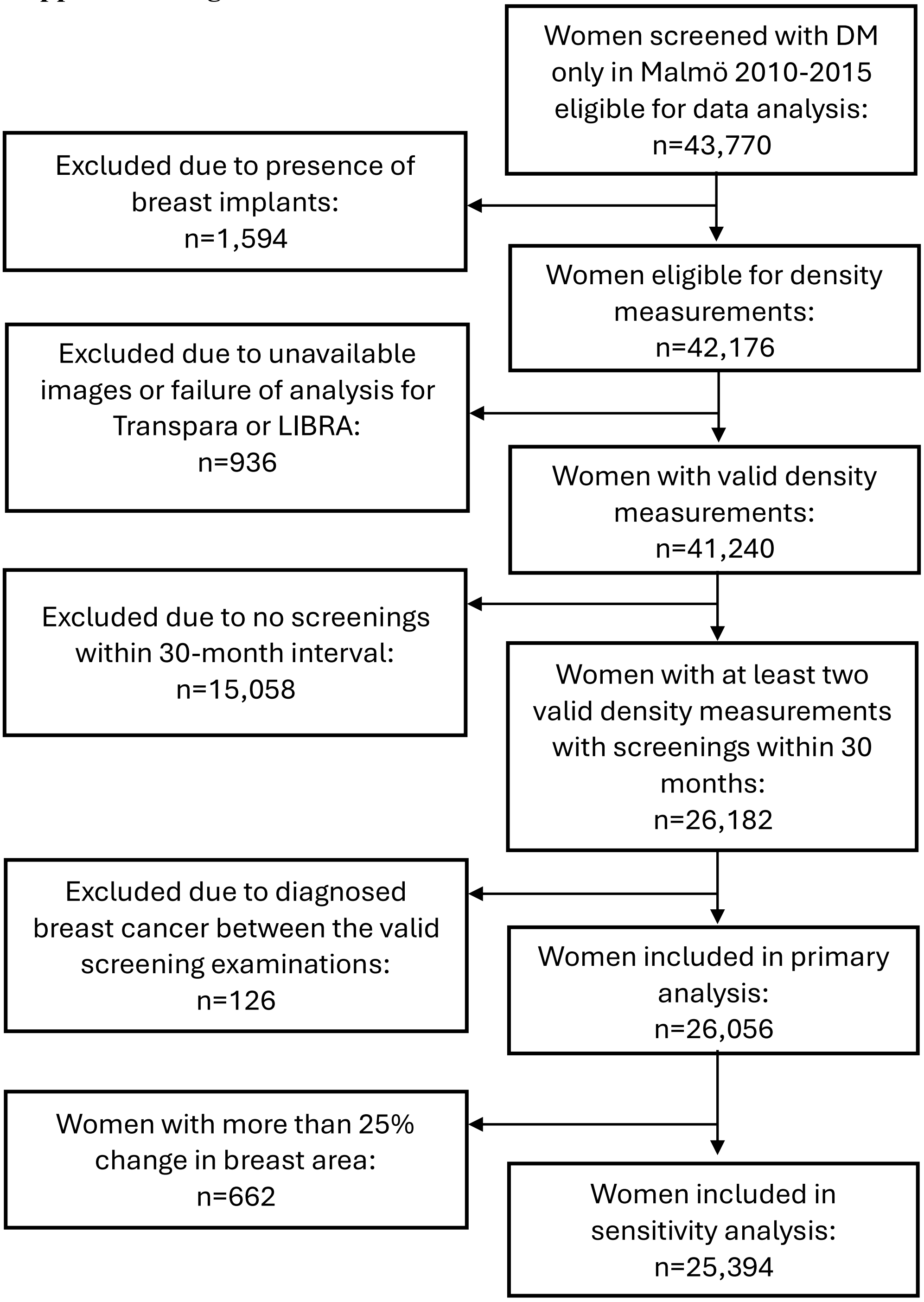


**Supplemental figure 1. Flowchart of study inclusion for sensitivity analysis**
Flowchart of population in the study with exclusions of examinations with a breast area change of more than 25% between the two screening examinations. DM = Digital Mammography. LIBRA= The Laboratory for Individualized Breast Radiodensity Assessment.

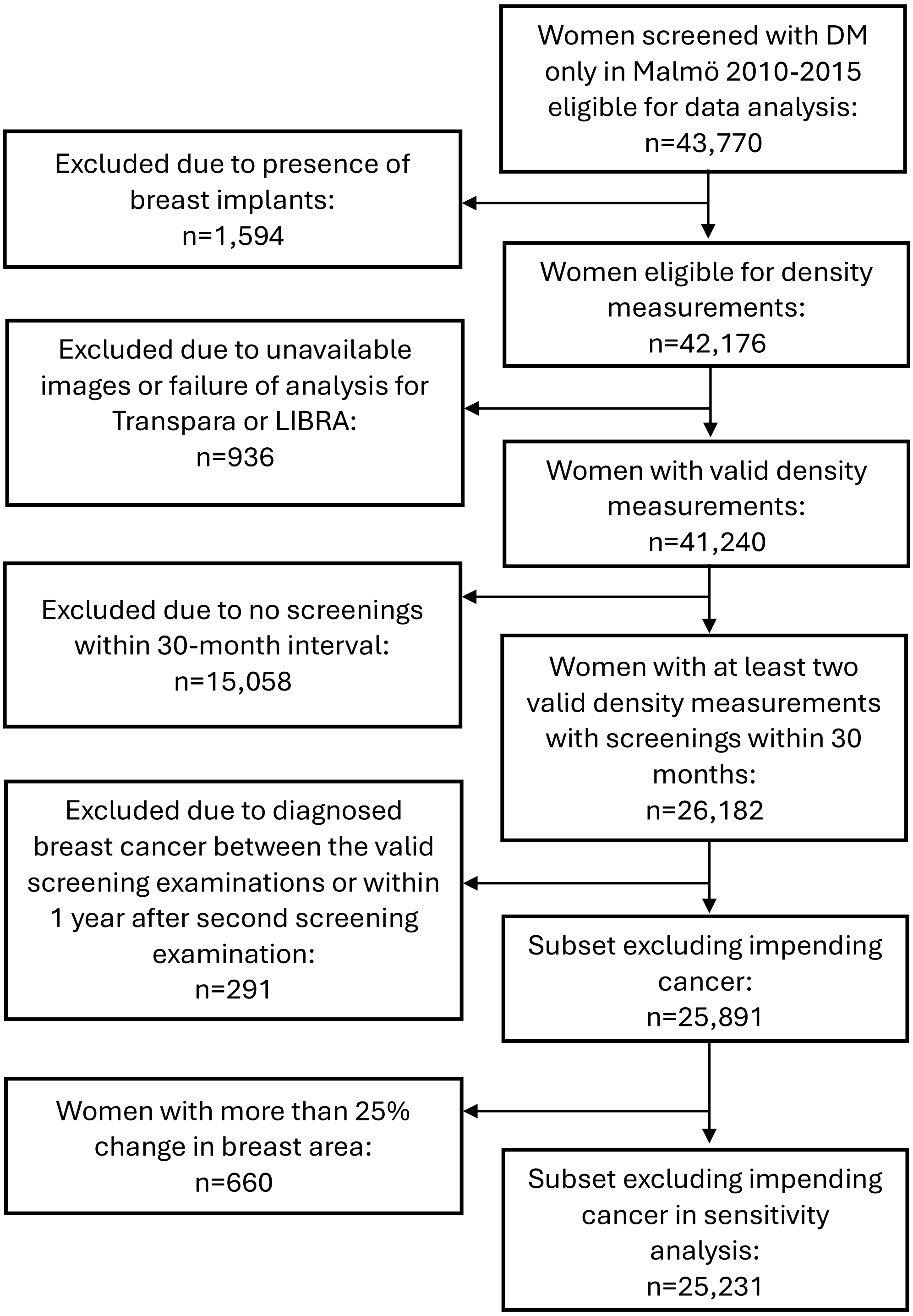


**Supplemental figure 2. Flowchart of study inclusion for sensitivity analysis with exclusion of women with impending cancer diagnosed up to one year after the second screening examination**

Flowchart of population in the study with exclusions of examinations with a breast area change of more than 25% between the two screening examinations when excluding women with impending cancer diagnosed up to one year after the second screening examination. DM = Digital Mammography. LIBRA= The Laboratory for Individualized Breast Radiodensity Assessment.

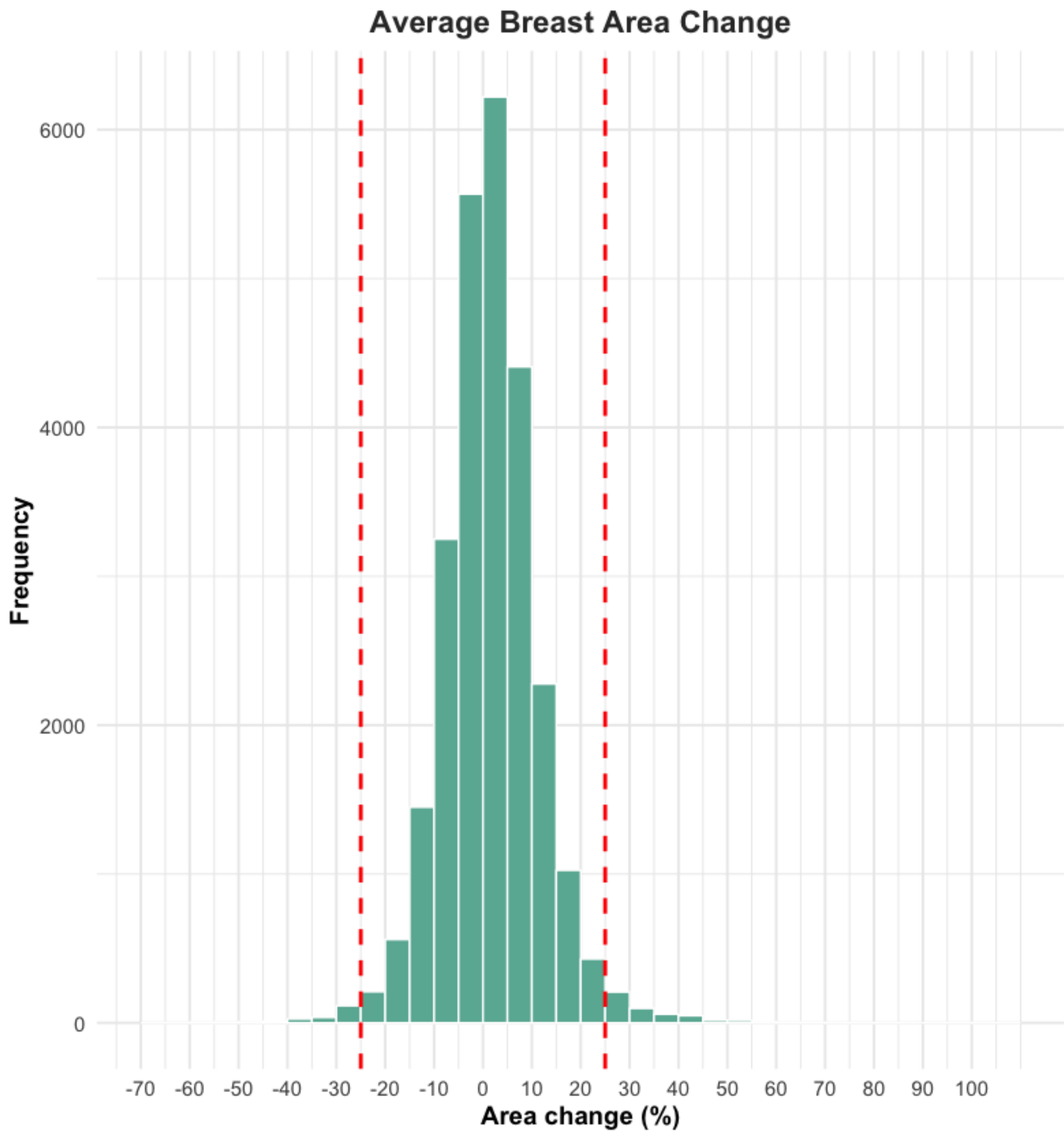


**Supplemental figure 3. Histogram of average breast area change between screening examinations**

Average breast area changes between screening examinations in percentages (%). Red dashed lines visualize the selected 25% limit, which in the post-hoc sensitivity analysis was considered to be a normal variation in our material.

**Table S1. Table showing multiple linear regression of area-based breast percentage density change**

Results of multiple linear regression examining the change in area-based breast percentage density (PD) between two screening examinations used as a dependent variable. The model includes future cancer diagnosis, initial area-based PD, age groups, and the time between examinations as predictors. Unadjusted data for each independent variable is also presented for comparison. CI = Confidence interval.

| **Variable** | | **Unadjusted** | | **Adjusted** | |
|---|---|---|---|---|---|
| | | **Estimate (95% CI)** | **P-value** | **Estimate (95% CI)** | **P-value** |
| Future cancer | | | | | |
| | Yes | 0.24 (-0.16, 0.64) | .25 | 0.50 (0.11, 0.88) | .01 |
| Initial area-based PD | | | | | |
| | Percentage | -0.09 (-0.10, -0.09) | <.001 | -0.10 (-0.11, -0.10) | <.001 |
| Age groups | | | | | |
| | Age 40-44 | - reference | - | - reference | - |
| | Age 45-54 | -0.68 (-0.87, -0.50) | <.001 | -1.55 (-1.73, -1.37) | <.001 |
| | Age 55-74 | 0.45 (0.27, 0.63) | <.001 | -0.78 (-1.01, -0.55) | <.001 |
| Time between examinations | | | | | |
| | Months | 0.01(-0.01, 0.03) | .20 | -0.12 (-0.14, -0.09) | <.001 |

**Table S2 Table of multiple linear regression of area-based breast percentage density change for the subset excluding women with impending cancer diagnosed up to one year after the second screening examination**

Results of a multiple linear regression examining the change in area-based breast percentage density (PD) between two screening examinations used as dependent variable, for the subset excluding women with impending cancer diagnosed up to one year after the second screening examination. The model includes future cancer diagnosis, initial area-based PD, age groups, and the time between examinations as predictors. Unadjusted data for each independent variable is also presented for comparison. *Future cancer is defined as a cancer diagnosed at least one year after the second screening round up until the end of follow-up. CI = Confidence interval.

| Variable | | Unadjusted | | Adjusted | |
|---|---|---|---|---|---|
| | | Estimate (95% CI) | P-value | Estimate (95% CI) | P-value |
| Future cancer* | | | | | |
| | Yes | 0.07 (-0.37, 0.51) | .76 | 0.36 (-0.07, 0.78) | .10 |
| Initial area-based PD | | | | | |
| | Percentage | -0.09 (-0.10, -0.09) | <.001 | -0.10 (-0.11, -0.10) | <.001 |
| Age groups | | | | | |
| | Age 40-44 | - reference | - | - reference | - |
| | Age 45-54 | -0.68 (-0.87, -0.50) | <.001 | -1.55 (-1.74, -1.37) | <.001 |
| | Age 55-74 | 0.46 (0.28, 0.64) | <.001 | -0.79 (-1.02, -0.56) | <.001 |
| Time between examinations | | | | | |
| | Months | 0.01(-0.01, 0.03) | .17 | -0.12 (-0.14, -0.09) | <.001 |

**Table S3. Descriptive data of the study population in sensitivity analysis**

Descriptive data based on the first included screening examination are presented for the study population and for the subset excluding women with impending cancer diagnosed up to one year after the second screening examination (additional analysis), after exclusions of examinations with a breast area change of more than 25% between the two screening examinations. BI-RADS = Breast Imaging-Reporting and Data System. Area-based PD = Area-based breast percentage density. VBD% = Volumetric breast density percentage. ¤Follow-up time (months) presented as median with interquartile range.

| | | Full study population in sensitivity analysis Mean (SD) or Number (%) | Subset excluding impending cancer in sensitivity analysis Mean (SD) or Number (%) |
|---|---|---|---|
| Total number of participants | | 25,394 (100%) | 25,231 (100%) |
| Age at first screening | | 52.8 years (9.5) | 52.8 years (9.5) |
| Days between examinations | | 660 days (109) | 659 days (109) |
| Age groups | | | |
| | Age 40-44 | 6,819 (26.9%) | 6,794 (26.9%) |
| | Age 45-54 | 8,664 (34.1%) | 8,621 (34.2%) |
| | Age 55-74 | 9,911 (39.0%) | 9,816 (38.9%) |
| Future cancer | | | |
| | Yes | 877 (3.5%) | 714 (2.8%) |
| | No | 24,517 (96.5%) | 24,517 (97.2%) |
| Median follow-up time | | 111 months (20)¤ | 111 months (20)¤ |
| BI-RADS density category at first screening | | | |
| | A | 2,469 (9.7%) | 2,456 (9.7%) |
| | B | 10,537 (41.5%) | 10,471 (41.5%) |
| | C | 8,748 (34.4%) | 8,691 (34.4%) |
| | D | 3,640 (14.3%) | 3,613 (14.3%) |
| Area-based PD at first screening | | 27.5% (16.4) | 27.5% (16.4) |
| VBD% at first screening | | 10.6% (5.4) | 10.6% (5.4) |

**Table S4. Table showing breast density change over one screening interval in sensitivity analysis**

Mean area-based breast percentage density (PD) and volumetric breast density percentage (VBD%) from the first and second included screening examinations, after exclusions of examinations with a breast area change of more than 25% between the two screening examinations. The table includes number of days between examinations and the difference in density, in parentage points (pp), between them. CI = Confidence intervals using Student's t-distribution.

| | Days between examinations (95% CI) | Density first examination (95% CI) | Density second examination (95% CI) | Density change in pp (95% CI) | P-value using paired t-test |
|---|---|---|---|---|---|
| Area-based PD | 660 (658, 661) | 27.5 (27.3, 27.7) | 26.1 (25.9, 26.3) | -1.3(-1.4, -1.3) | <0.001 |
| VBD% | 660 (658, 661) | 10.6 (10.6, 10.7) | 10.2 (10.1, 10.3) | -0.4 (-0.5, -0.4) | <0.001 |

**Table S5. Table showing breast density change over one screening interval in sensitivity analysis for the subset excluding women with impending cancer diagnosed up to one year after the second screening examination**

Mean area-based breast percentage density (PD) and volumetric breast density percentage (VBD%) from the first and second included screening examinations, after exclusions of examinations with a breast area change of more than 25% between the two screening examinations and after excluding women with impending cancer diagnosed up to one year after the second screening examination. The table includes the number of days between examinations and the difference in density, in parentage points (pp), between them. CI = Confidence intervals using Student's t-distribution.

| | Days between examinations (95% CI) | Density first examination (95% CI) | Density second examination (95% CI) | Density change in pp (95% CI) | P-value using paired t-test |
|---|---|---|---|---|---|
| Area-based PD | 659 (658, 661) | 27.5 (27.3, 27.7) | 26.1 (25.9, 26.3) | -1.4(-1.4, -1.3) | <0.001 |
| VBD% | 659 (658, 661) | 10.6 (10.6, 10.7) | 10.2 (10.1, 10.3) | -0.4 (-0.5, -0.4) | <0.001 |

**Table S6. Table showing multiple linear regression of volumetric breast density percentage change in sensitivity analysis**
Results of a multiple linear regression examining the change in volumetric breast density percentage (VBD%) between two screening examinations used as dependent variable, after exclusions of examinations with a breast area change of more than 25% between two screening examinations. The model includes future cancer diagnosis, initial VBD%, age groups, and the time between examinations as predictors. Unadjusted data for each independent variable is also presented for comparison. CI = Confidence interval.

| **Variable** | | **Unadjusted** | | **Adjusted** | |
|---|---|---|---|---|---|
| | | **Estimate (95% CI)** | **P-value** | **Estimate (95% CI)** | **P-value** |
| Future cancer | | | | | |
| | Yes | 0.04 (-0.08, 0.17) | .51 | 0.13 (0.00, 0.25) | .04 |
| Initial VBD% | | | | | |
| | Percentage | -0.09 (-0.09, -0.09) | <.001 | -0.09 (-0.10, -0.09) | <.001 |
| Age groups | | | | | |
| | Age 40-44 | - reference | - | - reference | - |
| | Age 45-54 | -0.18 (-0.24, -0.13) | <.001 | -0.42 (-0.48, -0.36) | <.001 |
| | Age 55-74 | 0.22 (0.17, 0.28) | <.001 | -0.12 (-0.20, -0.05) | <.001 |
| Time between examinations | | | | | |
| | Months | 0.02(0.01, 0.02) | <.001 | -0.03 (-0.03, -0.02) | <.001 |

**Table S7. Table showing multiple linear regression of volumetric breast density percentage change in sensitivity analysis for the subset excluding women with impending cancer diagnosed up to one year after the second screening examination**

Results of a multiple linear regression examining the change in volumetric breast density percentage (VBD%) between two screening examinations used as dependent variable, after exclusions of examinations with a breast area change of more than 25% between the two screening examinations and after excluding women with impending cancer diagnosed up to one year after the second screening examination. The model includes future cancer diagnosis, initial VBD%, age groups, and the time between examinations as predictors. Unadjusted data for each independent variable is also presented for comparison. *Future cancer is defined as a cancer diagnosed at least one year after the second screening round up until the end of follow-up. CI = Confidence interval.

| Variable | | Unadjusted | | Adjusted | |
|---|---|---|---|---|---|
| | | Estimate (95% CI) | P-value | Estimate (95% CI) | P-value |
| Future cancer* | | | | | |
| | Yes | 0.04 (-0.10, 0.18) | .56 | 0.14 (0.00, 0.27) | .048 |
| Initial VBD% | | | | | |
| | Percentage | -0.09 (-0.09, -0.09) | <.001 | -0.09 (-0.10, -0.09) | <.001 |
| Age groups | | | | | |
| | Age 40-44 | - reference | - | - reference | - |
| | Age 45-54 | -0.18 (-0.24, -0.12) | <.001 | -0.42 (-0.47, -0.36) | <.001 |
| | Age 55-74 | 0.23 (0.17, 0.28) | <.001 | -0.12 (-0.19, -0.05) | <.001 |
| Time between examinations | | | | | |
| | Months | 0.02(0.01, 0.02) | <.001 | -0.02 (-0.03, -0.02) | <.001 |

**Table S8. Table showing multiple linear regression of area-based breast percentage density change in sensitivity analysis**

Results of a multiple linear regression examining the change in area-based breast percentage density (PD) between two screening examinations used as dependent variable, after exclusions of examinations with a breast area change of more than 25% between two screening examinations. The model includes future cancer diagnosis, initial area-based PD, age groups, and the time between examinations as predictors. Unadjusted data for each independent variable is also presented for comparison. CI = Confidence interval.

| Variable | | Unadjusted | | Adjusted | |
|---|---|---|---|---|---|
| | | Estimate (95% CI) | P-value | Estimate (95% CI) | P-value |
| Future cancer | | | | | |
| | Yes | 0.16 (-0.22, 0.55) | .41 | 0.42 (0.04, 0.79) | .03 |
| Initial area-based PD | | | | | |
| | Percentage | -0.09 (-0.09, -0.08) | <.001 | -0.10 (-0.10, -0.09) | <.001 |
| Age groups | | | | | |
| | Age 40-44 | - reference | - | - reference | - |
| | Age 45-54 | -0.67 (-0.85, -0.49) | <.001 | -1.47 (-1.65, -1.29) | <.001 |
| | Age 55-74 | 0.44 (0.27, 0.62) | <.001 | -0.69 (-0.91, -0.46) | <.001 |
| Time between examinations | | | | | |
| | Months | 0.01(-0.01, 0.03) | .16 | -0.11 (-0.14, -0.09) | <.001 |

**Table S9. Table showing multiple linear regression of area-based breast percentage density change in sensitivity analysis for the subset excluding women with impending cancer diagnosed up to one year after the second screening examination**

Results of a multiple linear regression examining the change in area-based breast percentage density (PD) between two screening examinations used as dependent variable, after exclusions of examinations with a breast area change of more than 25% between the two screening examinations and after excluding women with impending cancer diagnosed up to one year after the second screening examination. The model includes future cancer diagnosis, initial area-based PD, age groups, and the time between examinations as predictors. Unadjusted data for each independent variable is also presented for comparison. *Future cancer is defined as a cancer diagnosed at least one year after the second screening round up until the end of follow-up. CI = Confidence interval.

| Variable | | Unadjusted | | Adjusted | |
|---|---|---|---|---|---|
| | | Estimate (95% CI) | P-value | Estimate (95% CI) | P-value |
| Future cancer* | | | | | |
| | Yes | -0.04 (-0.47, 0.39) | .87 | 0.24 (-0.17, 0.66) | .25 |
| Initial area-based PD | | | | | |
| | Percentage | -0.09 (-0.09, -0.08) | <.001 | -0.10 (-0.10, -0.09) | <.001 |
| Age groups | | | | | |
| | Age 40-44 | - reference | - | - reference | - |
| | Age 45-54 | -0.67 (-0.85, -0.49) | <.001 | -1.48 (-1.66, -1.29) | <.001 |
| | Age 55-74 | 0.45 (0.27, 0.63) | <.001 | -0.69 (-0.92, -0.47) | <.001 |
| Time between examinations | | | | | |
| | Months | 0.01(-0.00, 0.03) | .13 | -0.11 (-0.14, -0.09) | <.001 |
